# A Parental Competence Scale: Dimensions and Their Association With Adolescent Outcomes

**DOI:** 10.3389/fpsyg.2021.652884

**Published:** 2021-04-15

**Authors:** Charo Reparaz, Sonia Rivas, Alfonso Osorio, Gabriela Garcia-Zavala

**Affiliations:** ^1^School of Education and Psychology, Universidad de Navarra, Pamplona, Spain; ^2^Institute for Culture and Society, Universidad de Navarra, Pamplona, Spain; ^3^IdisNA, Navarra Institute for Health Research, Pamplona, Spain; ^4^Department of Psychology, Universidad Católica San Pablo, Arequipa, Peru

**Keywords:** family, parenting, education, adolescence, values, parental competence

## Abstract

Positive family functioning during adolescence is usually studied analyzing parents' competences and children's relationship abilities. We present an instrument for the assessment of parental competence, which encompasses both educational style and transmission of values. The objective of the study was to analyze its factor structure and to assess its value in predicting adolescent outcomes. We recruited 2,459 high school students, aged 12–15, in 40 schools from Spain, Peru, Mexico, and Chile. They responded to the instrument and to other questions regarding lifestyles. Exploratory and confirmatory factor analyses were carried out in order to assess the internal structure of the instrument, and internal consistency of the resulting dimensions was calculated. Finally, regression analyses were performed to establish associations between the parenting dimensions and adolescent outcomes. Factor analyses showed a consistent structure, with good fit indices in the four countries. The final four factors include parenting styles (Warmth and Demandingness) and education in values (Fortitude and Privacy). Regression analyses showed associations with adolescent outcomes. For example, adolescents' life satisfaction was best predicted by Warmth and Education in fortitude. Practical implications are suggested.

## Introduction

The original educational function of the family has lost its power (Parada Navas, 2010). This loss of power is perceived in the problems presented within the family, mainly relating to disorganization (chaotic structure and dysfunctional communication) and to desertion of parenting functions (incompetence and neglect) (Torío López et al., 2015). Faced with this change, current educational research demands a universal revalorization of parental rearing.

A possible solution to this educational crisis can be found in family education, a construct which has been widely studied from diverse perspectives (Jones et al., 2013; Darling et al., 2014).

Family education can be defined and studied from each of its three main scenarios (Aguilar, 2005; Bernal et al., 2012): the education that happens within family life; the education aimed to empower family life; and the education for intervention in families. This empirical research is based on the analysis of the first scenario.

As we will see, empirical studies often focus on parents' educational styles, leaving aside the goal of such education: the values instilled in children. A review of existing instruments shows that the transmission of values is rarely considered. Given this lack, we find that it is necessary to create an instrument encompassing different issues: parental educational style, understood as a context variable (parental demandingness and warmth); attention to basic needs; and transmission of values through parenting practices (specifically, two specific goals in adolescent life: fortitude and privacy). Thus, based on Darling and Steinberg (1993)'s theory, and in their contextual model of parenting styles, through this work we present the proposal and validation of a new scale. Additionally, we will test whether or not the dimensions of the scale are associated with some adolescent behaviors.

Below we will begin to analyze the concept of parental competence and present a review of the main instruments used in research in this regard. Then we will present our proposal of instrument that assesses educational styles, parenting practices, and transmission of values.

## Parental Competence and Adolescence

Family education has opened up two main research lines: the study of parents' general characteristics (expressed in the analysis of parenting styles) and the study of specific parenting practices (specified in the parental competence construct).

On the one hand, parenting style has been defined as “a constellation of attitudes toward the child that are communicated to the child and that, taken together, create an emotional climate in which the parent's behaviors are expressed” (Darling and Steinberg, 1993, p. 488). The two classical theories that tackle the concept of parenting styles (interactive and joint construction models) continue to inspire research in the educational, psychological, and sociological fields (Torío López et al., 2008; Aroca and Cánovas, 2012). This has facilitated the development of programs to help parents, in order to promote positive relationships within the family.

On the other hand, parenting practices have been defined as “behaviors defined by specific content and socialization goals” (Darling and Steinberg, 1993, p. 492). The study of specific parenting practices has sought to uncover relationships between parents' behaviors and child development in different areas and educational phases (Kopala-Sibley et al., 2012; Nordahl et al., 2016; Bi et al., 2018; Gölcük and Berument, 2019). Likewise, scientific literature has attempted to analyze the different factors associated with parenting, separating its parts and components (Trepat et al., 2014; Hajal et al., 2015).

In short, the confluence of these two research lines (parenting styles and specific parenting practices) can be found in the proposal presented by Darling and Steinberg (1993). According to them, the process through which parenting practice influences child development is complex. In order to understand it properly, we need to take into account three different aspects of parenting: the goals pursued by socialization, the parenting practices used by parents to help the child reach those goals, and the parenting style (or the emotional climate in which this socialization occurs). These authors consider parenting styles as a contextual variable which moderates the relationships between parents' specific parenting practices and children's development outcomes.

Thus, parental competence has been defined as “the practical abilities parents have to nurture, protect, and educate their children, and to ensure them a sufficiently healthy development” (Barudy and Dantagnan, 2010, p. 34). This is considered a key element within positive parenting (Martínez-González et al., 2016). Following this concept, a promising research line has been launched within the educational field of programs and interventions, aiming to empower parents across child developmental phases by promoting knowledge, abilities, and attitudes that enhance the parental role (Martínez-González et al., 2016; Rodrigo et al., 2018; Lindhiem et al., 2020).

Parental competence has been studied at different stages of the life cycle: childhood (Townshend et al., 2016; Windhorst et al., 2019) and adolescence or emerging adulthood (Dittman et al., 2016; Huey et al., 2020). It has also been studied from different points of view: from parents' perception of their own performance (Stattin et al., 2015; Hsieh et al., 2019; Windhorst et al., 2019), from the child's perspective experiencing parental competence (De Los Reyes and Ohannessian, 2016), or from both perspectives (Janssens et al., 2015). Finally, this construct has mainly produced proposals from a palliative point of view (Gagnon et al., 2020; Zhou et al., 2020), and not so much from a preventive one. That is to say, proposals seek to empower families from a perspective of deficit rather than from a position of proactivity (Rodrigo et al., 2015).

In relation to the objectives of the study, Iarocci and Gardiner state that analyzing parental competence during the child's adolescence is crucial, since “this critical period constitutes an especially salient time for examining social competence, as rapidly maturing developmental capacities have significant implications in terms of adolescents' rapidly emerging new social-cognitive resources, changing social goals, and shifting social focus” (Iarocci and Gardiner, 2015, p. 216).

It is also relevant to study the teaching of social values by parents, because adolescents' social dimension is a part of their psychological adjustment, and it appears to predict future adulthood competences (Allemand et al., 2019; Calandri et al., 2019; Bloom and Lambie, 2020). Social values are understood as “criteria shared by the majority of a society in an ideal sense, all of which lead them to the better society. Societal values regulate and organize daily life” (Türkkahraman, 2014, p. 635). There are many social values and many classifications of them, but we decided to choose two of them because of their relevance during adolescence: fortitude and respect of privacy.

In this regard, one of the positive adjustment indicators studied in adolescence has been resilience (Ruvalcaba-Romero et al., 2016). Educating in resilience implies educating in fortitude, which first demands the parents' ability to teach their children to resist harmful influences and endure nuisances, in order to emerge stronger from problematic situations (Isaacs, 2010). Furthermore, this author indicates that educating in fortitude also implies teaching children to face their goals with firmness, initiative, constancy, and decision. Therefore, parents' needs go beyond acquiring a set of competences, and include practicing values such as prudence and justice, self-confidence, sensitivity toward personal conditions, knowledge of a unique relationship, and orientation toward maturity with patience and perseverance (Palet, 2007). These competences should be present first in them (parents), in order to be efficient (Palet, 2000).

Another indicator is respect for privacy. Within the family, the child learns (implicitly or explicitly) which issues belong to an individual's private sphere, and what level of privacy or diffusion must be given to information about oneself or about others. This is what we call education in privacy or intimacy. Educating in privacy demands an ability to teach children the value of their own privacy and to respect that of others (Isaacs, 2010). This is especially relevant during adolescence, when many new relations with others are established, both online and face-to-face, and because this is a difficult period to tackle (Zou and Wu, 2019). The study of this issue is critical, as respect for privacy is a family protective factor against addictions (Awaluddin et al., 2019).

It seems therefore necessary that parental competence includes the dimension of education in social values.

## Review of Instruments

Below we present a review of instruments measuring parental competence used in research. The main classification criterion has been whether the instrument assesses the two classical parenting styles dimensions (warmth/support and control) or whether it analyses other characteristics such as adaptability, cohesion, and feelings of efficacy.

Among the instruments measuring parenting styles dimensions, some focus on the *control* dimension. The *Escala de Percepción de Control Psicológico* [Psychological Control Perception Scale] (Fernández de Ortega, 2005) measures Psychological control, which refers to intrusive parental practices employed to influence the child's behavior. The *Escala de Percepción del Control Parental de Niños* [Scale of Children's Perception of Parental Control] (Betancourt and Andrade, 2007) has a wider scope, and measures different types of parental control.

In contrast, other instruments focus on *warmth*. The *Parental Acceptance-Rejection Questionnaire* [PARQ] (Rohner and Khaleque, 2005) concentrates on acceptance and rejection, though there is an extension (the PARQ/C) which adds a measure of parental control.

However, most instruments measure both warmth and control, following the framework suggested by Baumrind (1967) and by Maccoby and Martin (1983).

According to a recent systematic review, while warmth is measured homogeneously across instruments, control is assessed in very different ways (González-Cámara et al., 2019). In the original framework, control meant supervision and rule setting (which has been later called behavioral control, and has been associated with positive outcomes). Later, however, some measures have included psychological control (with negative outcomes). Some instruments evaluate both behavioral and psychological control: such is the case in the *Parenting Styles Index* [PSI] (Steinberg et al., 1992), the *Parenting Style Inventory II* [PSI-II] (Darling and Toyokawa, 1997), the *Parental Support Inventory* (Barber and Thomas, 1986), the *Adolescent's Perception of Parental Behavior Questionnaire* [APPBQ] (Lakshmi, 2002) or the *Escala para la Evaluación del Estilo Parental* [EEEP, Scale for the Evaluation of the Parenting Style] (Oliva et al., 2007). On the other hand, some other instruments have only a single measure of control, which may study either behavioral or psychological control. This is the case in the *ESPA-29* (Musitu and García, 2001), which measures overprotection, coercion, and physical punishment. The aforementioned PARQ/C has a control dimension which assesses the presence of an exaggerated behavioral control. These dimensions, like psychological control, have been associated with worse outcomes in adolescent children (González-Cámara et al., 2019).

Partially based within the classic framework, but with a different methodological approach, we find the *Parental Authority Questionnaire* [PAQ] (Buri, 1991) and its short version (Alkharusi et al., 2011). These instruments transform Baumrind's three styles (authoritarian, authoritative, and permissive) into three independent dimensions.

Some instruments have sought to measure parental competence by analyzing family functioning or adaptability. *The Family Assessment Device* [FAD] (Epstein et al., 1983) assesses general functioning, problem solving, communication, roles, affective responsiveness, affective involvement, and behavior control. The Family Adaptability and Cohesion Evaluation Scale [FACES] (Olson, 2011) assesses family cohesion and adaptability.

Other instruments focus on the feelings of efficacy and satisfaction in parents and in other family members. In this regard, we may refer to the *Escala de Evaluación Parental* [Parental Evaluation Scale, EEP] (Farkas-Klein, 2008), for mothers of children under 2 years of age; the *Self-Efficacy for Parenting Tasks Index* [SEPTI] (Coleman and Karraker, 2000), for parents with children in Primary Education; and the *Escala de Satisfacción Familiar por Adjetivos* [Adjective-based Family Satisfaction Scale, ESFA] (Barraca and López-Yarto, 2017).

### Beyond Climate and Practices: Promotion of Values

The above instruments measure formal elements regarding the way in which parents behave. However, they do not tackle the issue of which values parents promote in their children.

Certain instruments exist that assess values in children. Such is the case with the *Scale for Preschool Social Values Acquisition* (Atabey and Ömeroglu, 2016), the *Multi-Component Gratitude Measure* [MCGM] (Morgan et al., 2017), the *Moral Values Internalization Questionnaire* [MVIQ] (Hardy et al., 2008), and the *Francis Moral Values Scales* (Village and Francis, 2016). However, these instruments do not deal specifically with how (or whether) parents encourage these values in their children.

We have found one instrument that has advanced in this direction. This is the *Parenting Competence Scale for Parents with Young Children* [PCS-YC] (Martínez-González and Iglesias-García, 2018). This scale introduces a subscale named “Promotion of Children's Self-Esteem,” with items such as “I make my children feel they are able to make decisions, even if they are young.” However, this instrument refers to young children (rather than adolescents) and is completed by the parents themselves.

It is therefore necessary to ask adolescents about the overall education they received from their parents regarding issues that are particularly relevant during this developmental stage: education in social values, and a positive climate of both warmth and demandingness. That is to say, we should combine issues regarding parenting styles and the specific content of socialization during adolescence, into one single instrument.

Thus, we present a scale to assess parental competence as perceived by adolescents, including the parental educational style understood as the context (demandingness and warmth), family coexistence, and education in two values which are important during this stage: privacy and fortitude. We hypothesize that the instrument will show a structure similar to the theoretical proposal, with good fit indices and internal consistency measures, and that the dimensions regarding both parenting styles and education in values will predict adolescent outcomes in a protective direction.

## Method

Materials and details on the Methods can be obtained contacting the corresponding author. Below we describe our sample, all data exclusions, all manipulations, and all measures in the study.

### Sample

Participants were recruited through a convenience sample of high school students from Spain, Peru, Mexico, and Chile. Schools were invited through different means in different Spanish-Speaking countries (because the instrument is written in Spanish). In participating schools, students from the 8th grade were invited to respond to a questionnaire. Participation took place in 16 schools from Spain, 14 from Peru, five from Mexico, and five from Chile. Participants were made up of 2,459 students-−56.0% female, aged 12–15 (Mean = 13.3, *SD* = 0.69). Most participants (55.1%) were from private schools (Table 1). This sample size is considered more than enough for the analyses described below (Lloret-Segura et al., 2014).

**Table 1:** Main characteristics of participants.

**Characteristics**	**Spain**	**Peru**	**Mexico**	**Chile**	**Total**
	***n* (%)**	***n* (%)**	***n* (%)**	***n* (%)**	***n* (%)**
	**(*N* = 781)**	**(*N* = 813)**	**(*N* = 543)**	**(*N* = 322)**	**(*N* = 2,459)**
**Sex**					
Male	286 (36.6)	337 (41.5)	316 (58.2)	144 (44.7)	1,083 (44.0)
Female	495 (63.4)	476 (58.6)	227 (41.8)	178 (55.3)	1,376 (56.0)
**Age (years)**					
12	9 (1.2)	109 (13.4)	66 (12.2)	0 (0.0)	184 (7.5)
13	571 (73.1)	492 (60.5)	185 (34.1)	132 (41.0)	1,380 (56.1)
14	183 (23.4)	189 (23.3)	225 (41.4)	171 (53.1)	768 (31.2)
15	18 (2.3)	23 (2.8)	67 (12.3)	19 (5.9)	127 (5.2)
**School**					
Public/Semi-public	583 (74.7)	296 (36.4)	0 (0.0)	225 (69.9)	1,104 (44.9)
Private	198 (25.4)	517 (63.6)	543 (100.0)	97 (30.1)	1,355 (55.1)

### Instruments and Variables

#### The Family Education Scale (Escala de Educación Familiar, EEF)

The scale measures parental competence, as perceived by the adolescent child, and intends to uncover strong and weak points in parental competence.^1^

The original scale consisted of 62 items, divided into three areas. With the aim of analyzing content validity of the scale we held different meetings with experts, examining its content in detail. Furthermore, we ran a pilot study in a sample of 85 school students aged 13–15. Internal consistency values of the data obtained are presented in Table 2.

**Table 2:** Internal consistency of the scales in the preliminary study.

**Scale**	**No. of items**	**Cronbach's alpha**
Basic needs	5	0.549
Daily coexistence	8	0.853
Affection	8	0.852
Demandingness	17	0.654
Fortitude	11	0.844
Privacy	13	0.806

After obtaining these results, we proceeded to test the scale in the sample of the present study.

Annex I shows the definitive Scale, reduced to 35 items.

#### Adolescent Outcome Variables

The Family Education Scale was included in a broader multi-purpose questionnaire, which included other variables. Some of these were used in order to assess the association between parenting and adolescent outcomes.

Regarding School grades, participants had to choose among these options: (1) “I failed at least one subject”; (2) “I passed all subjects, with average grades”; and (3) “I passed all subjects, with good grades.” As the most frequent answer was the third option, the dichotomized variable grouped the first two answers into the “Bad grades” category.

Life satisfaction was assessed with four items such as “I generally feel free in my life” or “I am generally happy with the life I lead.” Participants responded with how often each statement applied to their lives, from 0 (never) to 4 (always). The mean of the four items was calculated, and then dichotomized by a median split. Internal consistency was very good (ordinal alpha = 0.82, *N* = 2,182).

Regarding alcohol use, participants were asked: “During the last 12 months, how often have you… Consumed alcoholic beverages?” Possible answers ranged from “Never” to “3 days per week or more.” The variable was dichotomized: “Never” vs. all other responses (category labeled “Sometimes”).

Pornography use was treated similarly. The question was: “During the last 12 months, how often have you… Looked at erotic or pornographic material?” Possible answers ranged from “Never” to “3 days per week or more.” The variable was dichotomized: “Never” vs. all other responses (category labeled “Sometimes”).

Internet dependence was assessed with five items such as “I spend time absently, looking at my smartphone, tablet, or computer even when I could be doing more productive things” or “At all times, I look at and answer emails, chats, and tweets, even when doing so interrupts other activities.” Participants had to indicate to what extent they agreed with each statement, from 0 (Totally disagree) to 4 (Totally agree). The mean of the five items was calculated, and then dichotomized by a median split. Internal consistency was good (ordinal alpha = 0.71, *N* = 2,219).

### Procedure

This study was framed within a broader project which studies adolescents' lifestyles and relationships (YourLife Project, http://proyectoyourlife.com/). Schools were invited to participate via email and referred to the project's website. The website describes the project and includes the instructions to participate. Schools willing to participate registered via the website and were given participation links (one for each participating grade in the school). Schools managed parental permission according to local policies.

Students were informed about the study 2 days before it took place, and again just before responding to the questionnaire. The questionnaire was completed online. Students were told that participation was voluntary and anonymous, and that they were able to decline participation or to leave all questions unanswered. They were also told that, by clicking “START,” they consented to participate in the survey.

All the variables assessed were included in a single questionnaire, together with other variables. Filling the whole questionnaire took around 30 min.

Ethical permission was granted by the Research Ethics Committee of the University of Navarra.

### Analyses

Participants out of the age rank (12–15) were excluded, and they are not included in the sample described above. In addition, in each analysis, participants with missing values in the key variables were excluded.

Analyses were performed with Stata, v. 12.0.

#### Factor Structure of the Instrument

##### Exploratory Factor Analysis

In order to perform Exploratory Factor Analysis (EFA) and Confirmatory Factor Analysis (CFA) in different samples, we randomly divided the Peruvian sample in two halves (we chose the Peruvian sample as it was the largest one). All EFAs were performed with the first Peruvian subsample. CFAs were performed with the second Peruvian subsample and with the samples of the remaining countries.

First, we checked the sufficiency of the sample size (*N* = 402) and the appropriateness of the data matrix for EFA (through the Bartlett test of sphericity and the KMO Measure of Sampling Adequacy). Then, we performed EFA, following recommendations from Lloret-Segura et al. (2014). We chose the iterated principal factor method and Promax oblique rotation because other instruments have found correlations between some of these measures (Steinberg et al., 1994; Oliva et al., 2007). Items with low loading (<0.4) were dropped one by one, starting with the ones with the lowest Communalities. Items with high loadings in more than one factor were also dropped.

With the resulting factors, internal consistency was assessed through Cronbach alphas. When a factor included too many items (12 or more) and alpha was high enough, we dropped the items with the lowest item-rest correlations, one by one (if the alpha value did not suffer a relevant decrease).

##### Confirmatory Factor Analysis

The resulting factor structure was tested through CFA. First, it was tested in the second Peruvian subsample. One model was tested with one single factor. Then, a model with the four factors obtained from the EFA, unrelated. Finally, a model with those four factors, with correlations among them. We compared the fit indices of the three models, and found that the third model had the best indices.

After this, we added possible covariance between pairs of items, as suggested by modification indices—but only if the covariance made sense in the theoretical perspective. In each step, we checked whether the fit indices had been improved. The fit indices used were the Root Mean Square Error of Approximation (RMSEA), the Comparative Fit Index (CFI), and the Standardized Root Mean Square Residual (SRMR).

When the best possible model was achieved, we tested that same model in each of the other countries.

##### Internal Consistency

Cronbach's alphas were calculated for each subscale, and for each country. In accordance with Nunnally (1978), values over 0.7 were considered acceptable.

#### Predictive Power of the Instrument. Association With Adolescent Outcomes

Finally, we tested the association between the four parenting scales and some adolescent outcomes. Outcome variables were dichotomized. Then we performed logistic regressions in which the parenting dimensions were the independent variables and the adolescent outcomes were the dependent variables.

## Results

### Factor Structure of the Instrument

#### Exploratory Factor Analysis (EFA)

With the first half of the Peruvian sample (*N* = 407), we performed EFA. First, we tested data adequacy to factor analysis. Bartlett's test of sphericity was significant (0<0.001), and the KMO measure was excellent (0.920). With regard to sample size, the 407 participants of this sample were more than enough according to recommendations (Lloret-Segura et al., 2014).

Parallel analysis suggested that five factors should be retained. However, the fifth factor had only three items with sufficient factor loadings (>0.45). As a result, we indicated that four factors should be retained. Then, we applied Promax oblique rotation. Items with low loading in all factors were eliminated one by one, in increasing order or communality. Finally, an item with high loadings in two factors was eliminated. The resulting factors were labeled Warmth, [Education in] Fortitude, [Education in] Privacy, and Demandingness. Table 3 shows factor loadings for each item in each factor.

**Table 3:** Rotated factor loadings for the final EFA solution.

**Original items in Spanish**	**English translation**	**Warmth**	**Fortitude**	**Privacy**	**Demandingness**
Te conocen bien y te entienden	Know and understand you well	0.6928			
Conocen a tus amigos	Know your friends	0.5260			
Se preocupan de que seas feliz	Care about you being happy	0.5707			
Supervisan tus tareas escolares	Supervise your homework	0.4447			
Se divierten contigo	Have fun with you	0.7727			
Comparten el tiempo libre contigo	Share free time with you	0.8797			
Te ayudan con tus estudios cuando lo necesitas	Help you with your studies when you need it	0.7317			
Hablan contigo de tus intereses (tus aficiones, las cosas que te gustan…)	Speak with you about your interests (hobbies, things you like.)	0.7232			
Te gustaría parecerte a ellos en las cosas importantes	You'd like to be like them on important things	0.6052			
Te dan ejemplo	Set an example to you	0.6003			
Te escuchan	Listen to you	0.8322			
Tienen en cuenta tus opiniones a la hora de hacer planes	Take your opinions into account when making plans	0.7287			
Te hablan con amabilidad	Speak with you kindly	0.6029			
Te ayudan cuando te sientes inseguro	Help you when you feel insecure	0.6592			
Sientes que te quieren, que te aceptan como eres	Make you feel that they love you, that they accept you as you are	0.5801			
Con ellos te sientes consolado y apoyado	You feel comforted and supported when you are with them	0.6732			
Sientes que tus cosas les interesan	You feel they are interested in you	0.7728			
Tienen tiempo para hablar contigo	Have time to talk with you	0.8021			
Se esfuerzan por estar contigo y ayudarte	Try to be with you and to help you	0.7685			
Te explican por qué tienes que hacer lo que te mandan	Explain why you have to do what they command you				0.5473
Te explican cómo tienes que hacer lo que te mandan	Explain how you have to do what they command you				0.5596
Te exigen cumplir un horario en casa	Require you to follow a schedule at home				0.5235
Deciden contigo lo que creen que tienes que hacer	Decide with you what they believe you have to do				0.5884
Limitan lo que gastas	Limit what you spend				0.5730
Limitan el tiempo en el que puedes ver la tv	Limit the time you may watch tv				0.6665
Te controlan el uso del móvil, internet	Control your use of the cell phone and the internet				0.4265
Te controlan los libros y las revistas	Control the books and magazines you read				0.4768
Te enseñan a ver el lado positivo de las cosas	Teach you to see the positive side of things		0.4772		
Te enseñan a no quejarte por cualquier cosa	Teach you not to complain about everything		0.7366		
Te enseñan a rechazar los caprichos	Teach you to avoid being fussy, picky		0.7234		
Te enseñan a mejorar, lograr tus objetivos	Teach you to improve, to achieve your goals		0.6182		
Te enseñan a no hacer algo solamente porque lo hacen los demás	Teach you not to do something only because others do it		0.6544		
Te enseñan a expresar tu opinión	Teach you to give your opinion		0.5869		
Te enseñan a tener iniciativa	Teach you to take initiative		0.6178		
Te enseñan a escuchar las ideas de los demás	Teach you to listen to others people's ideas		0.5887		
Te animan a no contar tus problemas y sentimientos a personas que no sean de confianza	Encourage you not to share your problems and feelings with people who are not trustworthy			0.4922	
Te animan a cuidar tu manera de vestir para no incomodar a los demás	Encourage you to dress appropriately so that you don't make others feel uncomfortable			0.4295	
Te animan a evitar ver imágenes o escuchar canciones con contenido sexual	Encourage you to avoid seeing pictures or listening to songs with sexual content			0.7061	
Te animan a dar importancia a la intimidad de tu cuerpo	Encourage you that the privacy of your body is important			0.7403	
Te animan a cuidar tu aspecto físico	Encourage you to take care of your physical appearance			0.5734	
Te animan a no obsesionarte con tu aspecto físico	Encourage you not to be obsessed about your physical appearance			0.6764	
Te animan a evitar mentir y fingir en los chats o en las redes sociales	Encourage you to avoid lying and pretending, in chats or social networks			0.7419	
Te animan a no dar información personal (tuya, de tus familiares o amigos) a otras personas a través de internet	Encourage you not to give personal information (yours, your families or your friends') to other people over the internet			0.7866	
Te animan a no sacar fotos, grabar conversaciones o publicar en internet cosas de otras personas sin permiso	Encourage you not to take pictures, record conversations, or post other people's stuff online without permission			0.7782	
Te animan a no hablar en público de cosas que conoces de amigos o de sus familias	Encourage you not to talk in public about things that you know about friends or their families			0.8613	

#### Confirmatory Factor Analysis (CFA)

Before testing the resulting EFA solution through CFA, we continued to eliminate some items. Certain factors had more items than necessary, and higher internal consistency than necessary. As such, in those factors, we eliminated the items with worse item-test correlations, while maintaining high internal consistency.

Then we tested the model in the second half of the Peruvian sample through CFA. The fit indices of the different models are shown in Table 4. Model 1 included one single factor, and had a poor fit. Model 2 included the four factors obtained in EFA, without any correlations among them. The fit was better but still poor. Model 3 permitted correlations between the factors, which improved the model fit. Then, modifications were made, one by one, in the correlations of items' error terms. These modifications were made when suggested by the software and when they made sense in conceptual terms. Finally, model 4 was achieved, with a very good fit for the Peruvian sample in use. We then tested the same model in the other samples, and found that the fit was good in all of them, proving configural invariance (or construct invariance) across countries.

**Table 4:** Fit indices in confirmatory factor analysis models.

**Model no**.	**Model name**	**Sample**	**χ^2^**	**df ^a^**	**χ^2^/df**	**RMSEA**	**SRMR**	**CFI**
1	1 factor	Peru 2^b^	3214.912	560	3.21	0.131	0.130	0.616
2	4 independent factors	Peru 2^b^	1723.327	560	1.72	0.087	0.258	0.832
3	4 related factors - initial	Peru 2^b^	1369.681	554	1.37	0.073	0.065	0.882
4	4 related factors - corrected	Peru 2^b^	1027.788	545	1.03	0.057	0.059	0.930
		Spain	1359.392	545	1.36	0.053	0.057	0.924
		Mexico	1159.102	545	1.16	0.054	0.047	0.929
		Chile	1026.269	545	1.03	0.067	0.061	0.897
		Boys	1307.484	545	1.31	0.048	0.045	0.942
		Girls	1673.362	545	1.67	0.051	0.049	0.935
		12–13 years	1623.765	545	1.62	0.048	0.046	0.943
		14–15 years	1396.923	545	1.40	0.055	0.049	0.928

a *Degrees of freedom*.

b *Second half of the Peruvian sample (the first half was used for exploratory factor analysis)*.

Additionally, we tested the model separated by sex, and by age groups. All groups achieved good fit indices with the same model.

#### Internal Consistency and Correlation Among the Subscales

Once the factor structure was confirmed in the four countries, we assessed internal consistency. Cronbach alphas were calculated and are shown in Table 5. Values were around 0.95 for Warmth, around 0.90 for Fortitude and Privacy, and between 0.70 and 0.76 for Demandingness.

**Table 5:** Internal consistency of the final scales, and correlations among them.

	**No. of items**	**Cronbach's alpha**				**Correlations ^a^**		
		**Peru**	**Spain**	**Mexico**	**Chile**	**Warmth**	**Fortitude**	**Privacy**
Warmth	11	0.948	0.953	0.943	0.960			
Fortitude	8	0.898	0.890	0.911	0.919	0.676		
Privacy	10	0.867	0.908	0.908	0.891	0.352	0.433	
Demandingness	6	0.701	0.761	0.746	0.701	0.305	0.301	0.257

a *Pearson correlation coefficient. All correlations were statistically significant (p < 0.001)*.

Table 5 also shows correlations among the subscales. All correlations were statistically significant. Fortitude had moderate correlations (between 0.4 and 0.7) with Warmth and with Privacy. The remaining correlations were weak (between 0.2 and 0.4).

### Predictive Power of the Instrument. Association With Adolescent Outcomes

Finally, we tested the association between the four parenting scales and some adolescent outcomes through logistic regressions, with the family education subscales as the independent variables, and adolescent outcomes as the dependent variables. The odds ratios (Table 6) were always statistically significant (*p* < 0.001 in most cases, *p* < 0.05 in all of them), and in the expected direction: parenting dimensions predicted better outcomes.

**Table 6:** Association between parenting dimensions and adolescent outcomes.

**Independent variables: family subscales**	**Dependent variables: adolescent outcomes**				
	**Alcohol use**	**Pornography**	**Internet dependence**	**School grades**	**Life satisfaction**
	**OR (95% CI)**^**a**^	**OR (95% CI)**^**a**^	**OR (95% CI)**^**a**^	**OR (95% CI)** ^**a**^	**OR (95% CI)** ^**a**^
Warmth	1.52 (1.34–1.73)^***^	1.19 (1.04–1.37)^*^	1.51 (1.37–1.67)^***^	1.24 (1.12–1.37)^***^	3.69 (3.19–4.26)^***^
Fortitude	1.40 (1.22–1.62)^***^	1.28 (1.11–1.48)^***^	1.51 (1.35–1.69)^***^	1.35 (1.21–1.52)^***^	2.50 (2.18–2.86)^***^
Privacy	1.24 (1.12–1.38)^***^	1.19 (1.07–1.32)^***^	1.20 (1.11–1.29)^***^	1.16 (1.07–1.25)^***^	1.35 (1.25–1.46)^***^
Demandingness	1.52 (1.32–1.76)^***^	1.32 (1.14–1.52)^***^	1.42 (1.29–1.56)^***^	1.12 (1.02–1.24)^*^	1.35 (1.23–1.49)^***^

a *Odds ratios (and 95% confidence intervals) of each adolescent outcome. In order to permit comparisons among odds ratios, variables were coded similarly: the good category (no alcohol or pornography use, no Internet dependence, good school grades, and high life satisfaction) was 1, and the bad one was 0. Therefore, all independent variables predict less alcohol use, less pornography use, less Internet dependence, higher school grades, and higher life satisfaction*.

* *p < 0.05*,

*** *p < 0.001*.

We also ran multiple logistic regression analyses (data not shown). Results were similar. The direction of the associations was still the same (family subscales associated with better outcomes), but statistical significance was lost in some cases.

We repeated these analyses for each country. The results (not shown) were also very similar.

## Discussion

### Factor Structure of the Instrument

According to the psychometric analyses, the proposed EEF has a structure that has good fit indices in the four countries.

First, we will discuss the differences between the original model and the final one obtained. Out of the six original factors (Satisfaction of needs, Family coexistence, Affection, Demandingness, Fortitude, and Privacy), the proposed model retains only four. Correlations among the items from the first three factors are high, with those from the Affection factor presenting the highest values, while some items from the other two factors were lost. As such, the reduction of these three factors (Satisfaction of needs, 5 items; Family coexistence, 8 items; Affection, 8 items) to one 11-item factor (which we labeled Warmth) is not problematic, and this allows us reduce the instrument—rendering it simpler.

The Demandingness factor retains the lowest number of items (6). We believe this may be due to two reasons. Firstly, the subscale included items referring to negative control (*They insult you; They beat you; They mock you*). Secondly, it included items that may have been perceived as ambiguous (*They let you do whatever you want; They let you watch anything on the internet; They let you watch any TV program*) or too long and difficult to interpret (*They give you orders, they tell you what you have to do; They explain you why you have to do what they order*). However, the resulting Demandingness factor relies on simple and direct questions (which are the ones which work the best) and focuses on behavioral control. As González-Cámara et al. (2019) found in a systematic review, it is common to find contradictory results in the literature regarding parental control. Instruments measure this dimension in different ways (psychological control, coercion, or behavioral control), and often do not indicate it explicitly. This is a complex construct with both positive and negative aspects. In our case, we are looking at demandingness or behavioral control and, as expected, this is associated with positive outcomes.

As we have explained above, we have not found any instruments which assessed parenting styles variables (warmth and demandingness) together with parental competence regarding education in social values during adolescence. As a result, we believe that the EEF is a significant contribution to the scientific community, as it represents an important step in comprehensively assessing parental competences as perceived by adolescents. Both dimensions (Education in fortitude and Education in privacy) were found to be adequately represented in two factors, which supports their specific role within the instrument.

Regarding the internal consistency of the subscales, we can compare results related to Warmth and Demandingness with previous studies (Education in Fortitude and in Privacy are new scales, difficult to compare with existing instruments). For example, we can compare them with some of the most frequently used instruments to assess these constructs (González-Cámara et al., 2019): the PSI (Steinberg et al., 1992, 1994) or the EEEP (Oliva et al., 2007). The internal consistency of our Demandingness measure was not very high (alpha = 0.70–0.76), but it was similar to the strictness/supervision scale of the PSI (alpha = 0.76) and to the Behavioral Control scale of the EEP (alpha = 0.76–0.78) (Oliva et al., 2007). In our Warmth subscale, however, the internal consistency indices (alphas over 0.94) were higher than in the acceptance/involvement scale in PSI (alpha = 0.72) or in the Affection/Communication scale in EEP (alpha = 0.88–0.90).

We can also comment on the small but significant correlation between our Warmth and Demandingness subscales (*r* = 0.305, *p* < 0.001). It was also similar to the one found between the corresponding subscales of the PSI (*r* = 0.34) (Steinberg et al., 1994) or between the Affection/Communication and the Behavioral Control scales of the EEEP: 0.26 for mothers and 0.39 for fathers (Oliva et al., 2007), or 0.34 for both parents together (Balaguer et al., 2021). On the contrary, measures of coercion or psychological control usually have negative correlations with warmth. This happens with the Psychological Control scale of the EEEP: *r* = −0.39 (mothers), −0.37 (fathers), and −0.35 (both parents together) (Oliva et al., 2007; Balaguer et al., 2021).

### Predictive Power of the Instrument. Association With Adolescent Outcomes

Next, we will discuss the association between the four dimensions of the instrument and the adolescent outcomes. Although warmth and demandingness are the two most commonly recognized axes of parental competence, this new instrument includes two other dimensions, given their important role in parental competence during adolescence: Education in fortitude and Education in privacy.

We observe that the four dimensions appear to be protective with regard to the various analyzed outcomes. As described above, adolescents whose parents score high in these dimensions consume less alcohol and pornography, have a lower internet dependence, obtain higher grades, and have higher life satisfaction. Furthermore, these associations were confirmed in the four studied countries. These results support the conclusions found in other studies. Below we will discuss the results for each dimension of the instrument.

In our study, the WARMTH dimension is that which best predicts Life satisfaction. This means that adolescents feel higher satisfaction if their parents listen to them, help them, are kind with them and enjoy activities with them. In this regard, Pérez-Fuentes et al. (2019), found that parents' warmth, communication, and humor explain adolescents' life satisfaction. Secondly, adolescents' perception of parental warmth also predicts lower frequencies of alcohol use. This is consistent with authors who assert that a lack of parental affection is a risk factor associated with alcohol use (González-Ramos et al., 2018). Thirdly, according to our results, Warmth is the variable that best protects adolescents from internet dependence. Similar results were found in studies with different populations (Karaer and Akdemir, 2019; Malander, 2019). Finally, Warmth was a good predictor of academic achievement. Previous studies have found affection to be associated with academic achievement (Rodríguez Rodríguez and Guzmán Rosquete, 2019; Moral-García et al., 2020) and with academic engagement (Reparaz and Sotés-Elizalde, 2019).

Regarding parental DEMANDINGNESS, our results show that this is principally related to Alcohol use and to Internet dependence. The association between demandingness, measured as parental monitoring and knowledge, and alcohol use among adolescents has also been found in previous research (Abdi et al., 2015; Pour et al., 2015; Osorio and González-Cámara, 2016; González-Ramos et al., 2018). The relationship between parental demandingness and internet dependence has also been found. Studies have shown that demandingness predicts less internet use, but only if that demandingness is not overprotective (Faltýnková et al., 2020) and if it is combined with warmth (Dingus Keuhlen et al., 2020). According to other studies, adolescents point out that their parents hardly control their use of Internet (Gairín Sallán and Mercader, 2018).

Another set of our results relate to the dimension concerning education in FORTITUDE. This construct is highly associated with Life satisfaction. We have not found studies measuring education in fortitude or education in resilience (a related construct, as explained above). However, resilience itself has been associated with life satisfaction (Liu et al., 2014). Other studies have found that resilience mediates between positive emotions (Cohn et al., 2009) or mindfulness (Bajaj and Pande, 2016) and life satisfaction. Our results also show a strong association between Fortitude and Internet dependence. Adolescents who acknowledge that their parents teach them to see the positive side of issues, to avoid complaints, and to have initiative are less likely to present internet dependence. For years, scientific literature has shown that adolescents' internet dependence is a growing problem. Li et al. (2010) found that resilience is negatively associated with internet dependence, and they suggest that this trait should be promoted among adolescents. Similarly, Shen (2020) found similar results between resilience and excessive use of smartphones.

Pornography use also seems to be predicted by education in fortitude, according to our data. There is a growing concern regarding children's and adolescents' exposure to pornography and its impact on their social and psychological development. Mesch (2009) found that frequent consumers of pornography on the internet differ from other users of the internet in some social characteristics. Specifically, they report lower degrees of commitment with family and of prosocial attitudes. Regarding the association between education in fortitude and academic achievement, our results are also consistent with previous research. Some studies have found that resilience predicts academic achievement (Deb and Arora, 2012; Novotn and Kreménková, 2016). It is reasonable to expect that education in fortitude would also predict this association, as we have found.

The dimension related with education in personal PRIVACY (in the sense of respecting one's own physical image and private issues and that of others) is the dimension most weakly associated with the outcomes. However, as with the other dimensions, it is associated with all outcomes in a significant and protective way. Adolescents who rated their parents highly in this dimension score better than their peers in the five outcomes analyzed.

Among our results, we find that education of adolescent children in privacy highly predicts better life satisfaction, lower alcohol use, and lower internet dependence. Similarly, Awaluddin et al. (2019) found that, when parents respect their children's privacy, they are less prone to internet addiction.

## Limitations

This study has some limitations. First, we used a convenience sample, which is not representative of the adolescent population in the studied countries. However, since the objective is not to estimate prevalences, this does not imply a serious problem. In addition, the fact that the same structure works in the four countries adds confidence to the generalizability of the results. Second, the study is cross-sectional. Therefore, the direction of causality between parenting dimensions and adolescent outcomes cannot be clearly established. However, in some cases such direction can be inferred. For example, the negative association between parental demandingness and alcohol use cannot be easy explained by reverse causation: it is not likely that, when the adolescents start drinking, their parents stop controlling them.

## Conclusion

On the one hand, this new instrument demonstrates an internal structure which is coherent with the theory, and which has good fit indices in the four countries. On the other hand, the resulting dimensions are associated, as expected, with adolescent outcomes. Both issues provide evidence of validity to the instrument.

## Further Implications

In this work, we have assessed parental competence from the point of view of adolescent children. However, the perception of family functioning varies according to the observer (De Los Reyes and Ohannessian, 2016). Therefore, a complete view implies the involvement of other informants (Hunsley and Mash, 2007). In this regard, it would be interesting to supplement this research with a parent's version of the scale, in order to identify possible discrepancies between the data of both groups. This could allow us to identify strong and weak points in educational practices that might be modified.

Likewise, it would be interesting to analyze differences according to the sex of parents (Lindfors et al., 2019) and children (Bo and Jaccard, 2020). This would help professionals in family guidance learn how to promote positive parenting within families.

As we have seen, there is growing evidence that family plays an important role in the promotion of healthy lifestyles among adolescents, since this is a key stage in which relevant aspects of adult life begin to be defined. As such, we highlight the need to focus research on parental competence programs with a wider scope. They should include values-based education and empowerment regarding life skills. This study has shown the relevance of strengthening these dimensions of adolescent education (respect to privacy and fortitude) as a part of parental competence.

Specifically, parents' needs in their educational role are different at each developmental phase because of the different influences by external (social pressure) and internal (believes and perceptions) factors. Adolescence is especially complex and defiant to families because external factors become more relevant and seem to question the previous educational work. This situation may make parents feel a certain lack of competence in their parenting role, to which it is necessary to respond offering recommendations and help. In this sense, our results may encourage to work along two lines:

- In the present, using this new instrument can provide families with a diagnosis of the adolescent's perception of parental competences. This diagnosis may help them make the right educational decisions in order to improve their work as educators. Moreover, family counselors may benefit from using this instrument to help their assistant work.
- In the future, it would be valuable to design a program to improve parenting competences in families with adolescent children, both in the preventive and in the proactive levels. This program might focus on two key issues during adolescence: fortitude and respect for privacy. This work with families should work in four aspects which have proven positive results (Morris et al., 2020): (a) exchange of information, (b) changing the perception of self-competence, (c) developing skills, and (d) solving applied problems.

## Data Availability Statement

The raw data supporting the conclusions of this article will be made available by the authors, without undue reservation.

## Ethics Statement

Ethical permission was granted by the Ethics Committee of the University of Navarra. Schools managed parental permission according to local policies. The participants were told that, by clicking “START,” they consented to participate in the survey.

## Author Contributions

CR and AO designed the study and performed the analyses. SR and GG-Z contributed to the conceptualization, review of literature and interpretation on results. All authors revised the draft, made substantial contributions, and approved the final manuscript.

## Conflict of Interest

The authors declare that the research was conducted in the absence of any commercial or financial relationships that could be construed as a potential conflict of interest.

## Footnotes

^1^In the original design of the scale, we had the collaboration of Aurora Bernal and the EASH team from the University of Navarra.
