# Supplementary material for: A Parental Competence Scale: Dimensions and Their Association With Adolescent Outcomes
Source: Front Psychol. 2021 Apr 15;12:652884. doi: 10.3389/fpsyg.2021.652884 (PMC8082951; doi:10.3389/fpsyg.2021.652884)
Supplement: Supplementary file 1 [file Table_1.pdf]

Final proposed scale (Spanish)

Escala de Educación familiar
INSTRUCCIONES

En la presente escala se recogen toda una serie de afirmaciones relacionadas con la forma en que los padres educan a sus hijos. Lee atentamente cada una de las frases (incluyendo el encabezado que aparece sobre cada bloque de preguntas) y valóralas de acuerdo con la siguiente escala:

Nada	Poco	Normal	Mucho	Todo
**Nunca**	**Casi nunca**	**A veces**	**Casi siempre**	**Siempre**
1	2	3	4	5

Marca con una X el valor numérico que corresponda a tu respuesta. No te detengas demasiado en cada pregunta, contesta de forma espontánea. Es muy importante que respondas a todas las preguntas.

¡Muchas gracias por tu colaboración!

Sexo: ❑ Varón ❑ Mujer Edad: _____ años

**Tus padres:**	Nunca Nada	Casi nunca Poco	A veces Normal	Casi siempre Mucho	Siempre Todo
1. Te dan ejemplo	1❑	2❑	3❑	4❑	5❑
2. Te controlan el uso del móvil, internet	1❑	2❑	3❑	4❑	5❑
3. Te conocen bien y te entienden	1❑	2❑	3❑	4❑	5❑
4. Te animan a evitar ver imágenes o escuchar canciones con contenido sexual	1❑	2❑	3❑	4❑	5❑
5. Sientes que te quieren, que te aceptan como eres	1❑	2❑	3❑	4❑	5❑
6. Te animan a cuidar tu aspecto físico	1❑	2❑	3❑	4❑	5❑
7. Te ayudan cuando te sientes inseguro	1❑	2❑	3❑	4❑	5❑
8. Te enseñan a tener iniciativa contando con los demás	1❑	2❑	3❑	4❑	5❑
9. Te animan a no sacar fotos, grabar conversaciones o publicar en Internet cosas de otras personas sin permiso	1❑	2❑	3❑	4❑	5❑
10. Te enseñan a expresar tu opinión y tus ideas	1❑	2❑	3❑	4❑	5❑
11. Te controlan los libros y las revistas	1❑	2❑	3❑	4❑	5❑
12. Te enseñan a ver el lado positivo de las cosas	1❑	2❑	3❑	4❑	5❑
13. Te animan a no contar tus problemas y sentimientos a personas que no sean de confianza	1❑	2❑	3❑	4❑	5❑
14. Te animan a dar importancia a la intimidad de tu cuerpo	1❑	2❑	3❑	4❑	5❑
15. Te escuchan	1❑	2❑	3❑	4❑	5❑
16. Te animan a cuidar tu manera de vestir para no incomodar a los demás	1❑	2❑	3❑	4❑	5❑
17. Se esfuerzan por estar contigo y ayudarte	1❑	2❑	3❑	4❑	5❑
18. Limitan lo que gastas	1❑	2❑	3❑	4❑	5❑
19. Tienen tiempo para hablar contigo	1❑	2❑	3❑	4❑	5❑
20. Te enseñan a mejorar, a lograr tus objetivos	1❑	2❑	3❑	4❑	5❑
21. Sientes que tus cosas les interesan	1❑	2❑	3❑	4❑	5❑
22. Te enseñan a no quejarte por cualquier cosa	1❑	2❑	3❑	4❑	5❑
23. Te hablan con amabilidad	1❑	2❑	3❑	4❑	5❑
24. Limitan el tiempo en el que puedes ver la televisión	1❑	2❑	3❑	4❑	5❑
25. Te enseñan a no hacer algo solamente porque lo hacen los demás	1❑	2❑	3❑	4❑	5❑
26. Te animan a no dar información personal (tuya, de tus familiares o amigos) a otras personas a través de Internet	1❑	2❑	3❑	4❑	5❑
27. Tienen en cuenta tus opiniones a la hora de hacer planes	1❑	2❑	3❑	4❑	5❑
28. Te animan a no hablar en público de cosas que conoces de amigos o de sus familias	1❑	2❑	3❑	4❑	5❑
29. Te exigen cumplir un horario en casa	1❑	2❑	3❑	4❑	5❑
30. Con ellos te sientes consolado y apoyado	1❑	2❑	3❑	4❑	5❑
31. Te animan a evitar mentir y fingir en los chats o en las redes sociales	1❑	2❑	3❑	4❑	5❑
32. Te enseñan a rechazar los caprichos	1❑	2❑	3❑	4❑	5❑
33. Deciden contigo lo que creen que tienes que hacer	1❑	2❑	3❑	4❑	5❑
34. Te enseñan a escuchar las ideas de los demás	1❑	2❑	3❑	4❑	5❑
35. Te animan a no obsesionarte con tu aspecto físico	1❑	2❑	3❑	4❑	5❑

Ítems: Exigencia parental: 2, 11, 18, 24, 29, 33. Afecto parental: 1, 3, 5, 7, 15, 17, 19, 21, 23, 27, 30. Educación de la fortaleza: 8, 10, 12, 20, 22, 25, 32, 34. Educación de la privacidad: 4, 6, 9, 13, 14, 16, 26, 28, 31, 35.
